# Labor Induction After Severe Preeclampsia With Maternal Posterior Reversible Encephalopathy Syndrome Complications Leading to Intrauterine Fetal Death: A Case Report

**DOI:** 10.7759/cureus.44250

**Published:** 2023-08-28

**Authors:** Xiaobin Fan, Jing Ning, Miao Zhang, Lu Gao, Hanyu Guo

**Affiliations:** 1 Obstetrics and Gynecology, The Affiliated Hospital of Northwest University, Xi'an No.3 Hospital, Xi'an, CHN

**Keywords:** prenatal period, intrauterine fetal death, induction of labor (iol), severe preeclampsia, posterior reversible encephalopathy syndrome (pres)

## Abstract

Posterior reversible encephalopathy syndrome (PRES) is a clinical imaging syndrome characterized by vasogenic edema in the posterior cerebral circulation, with severe preeclampsia (PE) and eclampsia as major etiologies. Posterior reversible encephalopathy syndrome lesions are often reversible, but they can be potentially fatal in obstetric crises, causing serious complications such as cerebral hemorrhage, confusion, headache, visual symptoms, and stroke if not treated immediately. Neurological sequelae and even death may occur in a minority of these cases.

In this paper, we report the case of a 26-year-old primigravida at 25 weeks of gestation who was irregular with obstetric visits. The patient presented with edema, nausea and vomiting, dizziness, blurry vision, falling down, and a maximum blood pressure of 190/85 mmHg. A brain MRI revealed PRES. Approximately 10 hours after admission, intrauterine fetal death occurred. After treatment, the patient was in stable condition and successfully induced for delivery.

## Introduction

Posterior reversible encephalopathy syndrome (PRES) was first proposed in 1996 and has a prevalence of 0.01% [1]. It is a multicausal, heterogeneous disorder characterized by a clinical imaging syndrome of vasogenic edema in the posterior cerebral circulating region of the brain [2]. Its diagnosis is becoming more common with the increasing availability of MRI [3]. Symptoms include seizures, impaired consciousness, headaches, and visual disturbances. Severe preeclampsia (PE) and eclampsia are the major causes, but it also occurs in patients suffering from bone marrow transplantation, chemotherapy, sepsis, and autoimmune disorders [4].

Preeclampsia is a clinical syndrome of new-onset hypertension with multi-organ or systemic involvement after 20 weeks of gestation, with a global prevalence of approximately 2% to 8%, and is one of the top two leading causes of maternal death worldwide [5]. Early recognition and standardized treatment are essential. Failure to do so may lead to permanent neurological deficits and, in severe cases, even death [6].

## Case presentation

The patient was a 26-year-old woman of 25 weeks of gestation, gravida 1, para 0, unmarried, with a regular menstrual cycle, and had not undergone any obstetric examination except a positive urine pregnancy test during her pregnancy until symptoms developed. She had no history of systemic disease. She initially complained of nausea and vomiting for three days. Nine hours before admission, she had dizziness with blurred vision and shortness of breath, without chest tightness or chest pain. Shortly thereafter, she became confused and fell several times outside the hospital, with mild pain and discomfort in her abdomen. The police at the roadside took her to the hospital. At the hospital, her blood pressure was up to 190/85 mmHg. Laboratory investigations demonstrated a significant elevation of white cell count (WBC), brain natriuretic peptide (BNP), proteinuria, and kidney function indicators (Table 1).

**Table 1:** Laboratory investigations on admission PT: prothrombin time; APTT: activated partial thromboplastin time; INR: international normalized ratio; FIB: fibrinolysis; BNP: brain natriuretic peptide; ALT: alanine aminotransferase; AST: aspartate aminotransferase; ALB: albumin; Cre: creatinine; BUN: blood urea nitrogen; LDH: lactate dehydrogenase

On admission	Day one	Day two	Day three	Day four	Day five	Day six
		19:45: Placed a Cook cervical ripening balloon	09:30: Ethacridine lactate amniocentesis; 19:55: Removed the Cook cervical ripening balloon; 23:00: Delivery			
Full blood count						
White blood cell count (×10^9^/L)	20.56	15.10	11.35	18.10	13.02	13.56
Hemoglobin (g/L)	115	91	81	83	76	71
Platelets (×10^9^/L)	171	128	118	123	131	145
Neutrophil% (%)	79.5	79.2	72	83.6	68.4	71.5
Lymphocyte% (%)	16.1	15	20.4	10.8	23.7	19.5
Coagulation function tests						
PT(s)	9.6	9.0	8.7	9.3	8.9	8.8
APTT (s)	24.9	27.6	29.7	29.8	28.4	28.4
INR	0.87	0.82	0.79	0.85	0.81	0.80
FIB (mg/dL)	417	384	369	411	359	332
D-dimer (ug/ml)	6840	18140	19965	24391	18590	5695
24-hour urine protein (mg)		4574				1002
BNP (ng/L)	207	258	321	266	178	
Liver function tests						
ALT (U/L)	42	28	31	25		12
AST (U/L)	60	55	68	45		21
ALB (g/L)	24.1	24.9	24.8	22.1		24.6
Kidney function tests						
Cre (umol/L)	118	105	93	75		57
BUN (mmol/L)	12.42	8.69	5.86	3.08		2.03
LDH (U/L)	599	431	382			244

The brain MRI scan showed diffuse bilateral cerebral edema of the frontal and occipital lobes. The cranial MR angiography (MRA), MR venography (MRV), diffusion-weighted imaging (DWI), and susceptibility-weighted imaging (SWI) proposed: 1. DWI: acute cerebral infarction in the corpus callosum pressure and bilateral occipital and parietal lobes; 2. SWI: diffuse microhemorrhages in the bilateral cerebral cortex; 3. MRV: narrow and localized signal discontinuity in the straight sinus, sinus sink, and bilateral transverse sinus; 4. MRA: diffuse signal heterogeneity and multiple stenosis changes in the intracranial arteries; combined with the above changes, reversible posterior encephalopathy syndrome (PRES) was considered.

**Figure 1:**
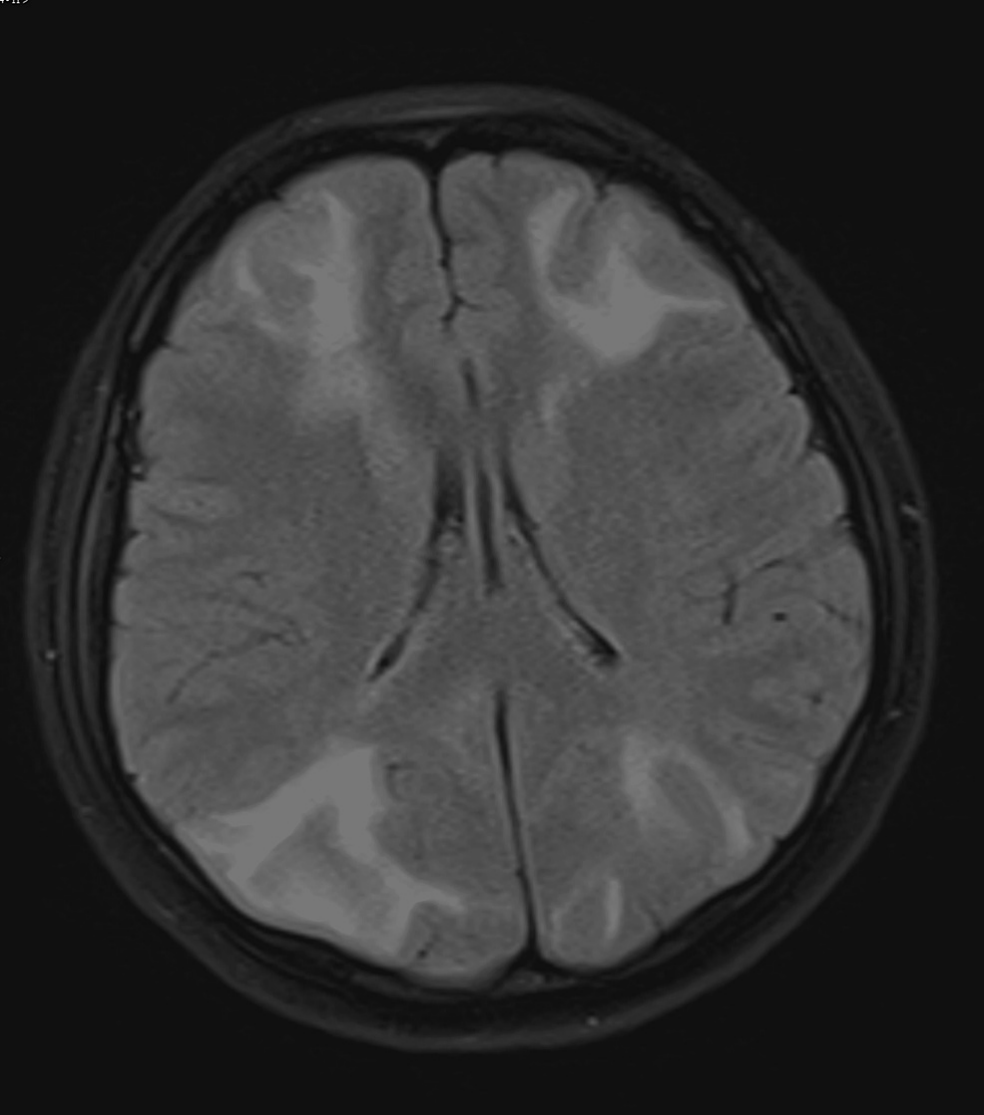
Magnetic resonance imaging (MRI) with T2 fluid-attenuated inversion recovery (T2 FLAIR) shows a symmetric hyperintense signal abnormality involving both the frontal lobes and bilateral occipital.

The diagnosis was early-onset severe preeclampsia (sPE), hypertensive encephalopathy, posterior reversible encephalopathy syndrome (PRES), partial hemolysis, elevated liver enzymes and low platelets (HELLP) syndrome, hypoproteinemia, and acute kidney injury. After admission, she was treated with an antihypertensive (labetalol 100mg every eight hours), an anticonvulsant, and neuroprotection (magnesium sulfate 5g intravenous loading dose, followed by a maintenance infusion of 1-2 g/h by a controlled infusion pump), correction of hypoproteinemia (human albumin 20g every day (Qd), acid suppression (omeprazole 40mg Qd), hepatoprotection (polyene phosphatidylcholine 456mg three times a day (Tid), and diuresis (furosemide 20mg Qd). The patient never planned to give birth. Initially, the fetal heart rate was 106 bpm, but intrauterine death occurred about 10 hours after admission. The patient's condition was stable.

Her Bishop score was one, and we chose the mechanical cervical ripening device with a Cook cervical ripening balloon before the patient slept. It is recommended to fill the upper uterine balloon (30 ml of saline) first. Then, gently pull the catheter and then fill the vaginal balloon (30 ml of saline), placing the balloons on either side of the cervix. Both balloons were then filled with a maximum of 80 ml of saline. The next morning, the patient received an intraamniotic injection of 100 mg of ethacridine lactate guided by ultrasound to induce labor. The Cook cervical ripening balloon was removed after nearly 24 hours. After three hours, the fetus was delivered successfully. The patient’s blood pressure gradually returned to normal without any symptoms after 10 days of delivery, and an MRI suggested a lamellar abnormal signal shadow in the right occipital lobe cortical area, which showed significant improvement, but the urine protein was still 3+, and she was instructed to follow up with the neurology and nephrology departments.

## Discussion

Posterior reversible encephalopathy syndrome (PRES) is often non-specific and often resolves when the causes are treated. Few cases have permanent neurological sequelae [7]. Therefore, PRES should be considered in pregnant women with PE and eclampsia who present with dizziness, headache, blurred vision, seizures, and disturbances of mental consciousness [8].

The pathogenesis of PRES has not yet been elucidated, and most of our knowledge comes from clinical and imaging data [9,10]. There are two hypotheses [11]. One is hyperperfusion, in which a rapid increase in blood pressure leads to dysregulation of the autoregulation of the cerebral vasculature, and hyperperfusion causes extravasation of plasma and macromolecules into the brain parenchyma, leading to vasogenic cerebral edema [12]. However, approximately 20%-50% of PRES cases have no history of hypertension [13]. Another hypothesis of endothelial dysfunction is that vascular endothelial damage from circulating endogenous or exogenous toxins deregulates the blood-brain barrier, causing severe cerebral vasoconstriction and hypoperfusion with cerebral edema [7,14]. As for PE and eclampsia, hypertension is the primary diagnostic criterion. Its pathophysiological mechanisms were vascular endothelial cell activation and dysfunction, intravascular inflammation, syncytiotrophoblast stress, and vasospasm [15,16]. Vasospasms may cause multi-organ and multi-system ischemia and hypoxia. Patients with PE are therefore at a higher risk of developing PRES [17].

In this case, the patient’s cranial MRI suggested the intracranial artery was narrowed in many places, which can cause circulatory ischemia, hypoperfusion, acute cerebral infarction, and bilateral diffuse micro hemorrhagic foci in the cerebral cortex, which may be due to a significant increase in the patient’s blood pressure. Hypertension can lead to ischemia after reperfusion and cause bleeding injuries, which are closely related to the prognosis of the patient. It can lead to intracranial hemorrhage, marked diffuse cerebral edema, and increased global intracranial pressure, resulting in neurological sequelae and even death. So, MRI is an important test for the assessment of changes in the patient's prognosis.

The patient’s laboratory index WBC was significantly elevated, which was considered to be more severe and related to an enhanced inflammatory or stress response because PE is essentially placental ischemia and hypoxia due to insufficient placental perfusion, and the cells are in a continuous state of oxidative stress, leading to increased release of pro-inflammatory cytokines Tumor necrosis factor alpha (TNF-α), interleukin-1, and IL-6, while the increase in intravascular toxic substances and inflammatory mediators causes endothelial damage and dysfunction [18]. In addition, patients have abnormally elevated levels of lactate dehydrogenase (LDH), uric acid, and creatinine, which are good indicators for monitoring the dysfunction of hemolysis and vascular endothelial cell damage and to assess the severity of the condition in pregnant women with hypertensive disorders of pregnancy. However, they are not specific laboratory indicators to predict PRES [19].

For PRES, there’s no specific treatment, but the definitive treatment for PE with severe features is timely delivery [7,20]. With the induction of labor, the likelihood of a cesarean section increases with increasing gestational age in patients with early-onset severe PE (range, 93%-97%＜28 weeks’) [21]. Fetal distress is the main reason. Induction of labor was preferred in this patient because of a combination of intrauterine fetal death and the avoidance of the long-term risks and complications associated with a cesarean section. The patient didn’t develop eclampsia, require admission to the intensive care unit (ICU), or even have neurological sequelae. Vaginal delivery may be a considerable option for very preterm elective termination or intrauterine fetal death with PRES [22].

## Conclusions

Posterior reversible encephalopathy syndrome is a clinical imaging syndrome of vasogenic edema of the posterior cerebral circulation that causes non-specific neurological symptoms and should be considered in pregnant women. It is important to recognize the various neurological symptoms, including seizures, headaches, altered mental status, and visual disturbances. Regardless of whether their blood pressure is elevated, these patients should be considered for PRES, and brain imaging should be performed for early diagnosis and treatment to avoid adverse outcomes such as neurological sequelae or death. Despite the fact that our knowledge of the PRES has grown, there is a need to enhance our management of antenatal care.
